# A Self-Oscillating Driving Circuit for Low-Q MEMS Vibratory Gyroscopes

**DOI:** 10.3390/mi14051057

**Published:** 2023-05-16

**Authors:** Tian Han, Guanshi Wang, Changchun Dong, Xiaolin Jiang, Mingyuan Ren, Zhu Zhang

**Affiliations:** 1 Department of Artificial Intelligence, Jinhua Advanced Research Institute, Jinhua 321013, China; htopen@foxmail.com (T.H.); hitdongcc@163.com (C.D.); jlynner@163.com (X.J.); rmy2000@126.com (M.R.); 2 Development Strategy Research Center, China Electronics Technology Group, Beijing 100846, China; wanggs@163.com

**Keywords:** MEMS, vibratory gyroscopes, self-oscillating driving circuit, automatic gain control, frequency modulation drive circuit

## Abstract

This article establishes a circuit model with which to analyze the difficulty of auto-gain control driving for low-Q micromechanical gyroscopes at room temperature and normal pressure. It also proposes a driving circuit based on frequency modulation to eliminate the same-frequency coupling between the drive signal and displacement signal using a second harmonic demodulation circuit. The results of the simulation indicate that a closed-loop driving circuit system based on the frequency modulation principle can be established within 200 ms with a stable average frequency of 4504 Hz and a frequency deviation of 1 Hz. After the system was stabilized, the root mean square of the simulation data was taken, and the frequency jitter was 0.0221 Hz.

## 1. Introduction

Over the last decade, micro-electromechanical system (MEMS)-based inertial sensors have undergone widespread developments in both research efforts and commercial products, offering advantages such as low cost, small size, low power consumption and suitability for batch fabrication [1,2,3,4].

In general, the gyroscopes are driven by AC voltage. This approach often involves utilizing an external oscillator to generate a driving signal that aligns with the resonant frequency of the mechanical gyroscope driving direction. To achieve frequency matching, the oscillator’s frequency is manually adjusted [5]. However, this method is often inaccurate, particularly when the gyroscope’s parameters fluctuate due to changes in the external environment. To tackle this issue, a closed-loop driving method has been introduced to facilitate automatic frequency matching between the driving circuit and the mechanical structure [6,7]. The benefit of this implementation method is that it averts frequency imbalances caused by structural parameter shifts resulting from variations in the external environment. As the quality factor of the micromechanical gyroscope structure is significantly high, it operates akin to embedding a narrowband band-pass filter function in the system [8]. The closed-loop drive system capitalizes on the frequency selection capability of the structure to achieve automatic tracking and matching of frequencies [9]. This filter significantly attenuates signals whose frequencies are dissimilar to the resonant frequencies of the structure drivers while amplifying signals whose frequencies are equivalent to this resonant frequency, culminating in the formation of a stable closed-loop driving signal [10].

In the gyroscope driving circuit, both the loop gain and the loop phase are related to the quality factor Q [11]. The Q value of the micromechanical gyroscope in the atmospheric environment is only a few hundred [12]. In this case, the loop gain of the circuit is large and other frequency signals in the environmental noise are also amplified, resulting in the frequency of the output signal of the gyroscope closed-loop drive circuit and the driving mode [13]. There is a deviation in the natural frequency of the state, and the frequency jitter is also large [14]. To resolve this problem, the micromachined gyro sensor structure is usually vacuum packaged to improve its Q value; however, this increases the manufacturing costs [15]. The traditional gyro closed-loop drive circuit is generally based on the principle of automatic gain control [16]. When driven by this principle, when the micromechanical gyro is in the working state, its structure is in a resonant state, and the driving voltage signal will be coupled with the driving direction detection terminal via parasitic capacitance [17]. It causes the amplitude and phase of the driving direction detection signal to change so that the self-excited driving conditions of the system cannot be satisfied [18].

This article presents a driving circuit that utilizes the frequency modulation principle to eradicate the identical frequency coupling from the driving signal to the displacement signal. Moreover, this circuit is harmonious with low-Q-value gyroscope structures.

This paper is organized as follows. Section 2 illustrates the MEMS gyroscope structure and principle. Section 3 illustrates the automatic gain control circuit. Section 4 describes the frequency modulation drive circuit and implementation details. The results of the simulation are presented and discussed in Section 5, and the conclusions are listed in Section 6.

## 2. MEMS Gyroscope Structure and Principle

**Figure 1:**
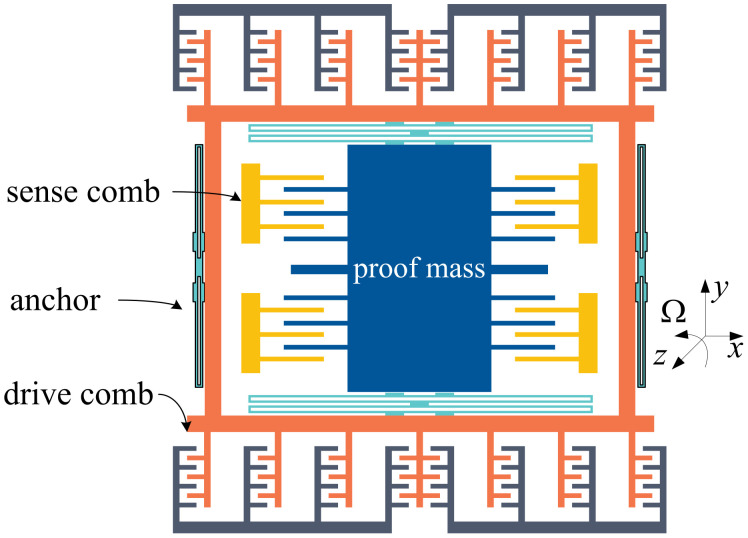
MEMS vibratory gyroscope structure.

MEMS vibratory gyroscopes are generally composed of a proof mass with elastic beams and corresponding combs in both directions for capacitance detection. Figure 1 shows a standard model of the sensitive structure for MEMS gyroscopes with a single proof mass driven in the x-direction [19].

The gyroscope operates in two distinct modes: driving and detecting. In the driving mode, a stable driving signal is added to the gyro structure, producing Coriolis force from the angular velocity signal in the *z*-axis direction. This displacement generates changes in the detection capacitance along the *y*-axis. In the detecting mode, the detected capacitance displacement (or angular velocity signal) is converted into a voltage signal output via the interface circuit [20].

### 2.1. Drive Mode

The closed-loop drive mode is commonly utilized in micromechanical gyroscopes. After undergoing processes such as peak detection and variable gain, the driving electrode’s vibration signal is fed back to the driving electrode to create a closed-loop drive signal [21].

**Figure 2:**
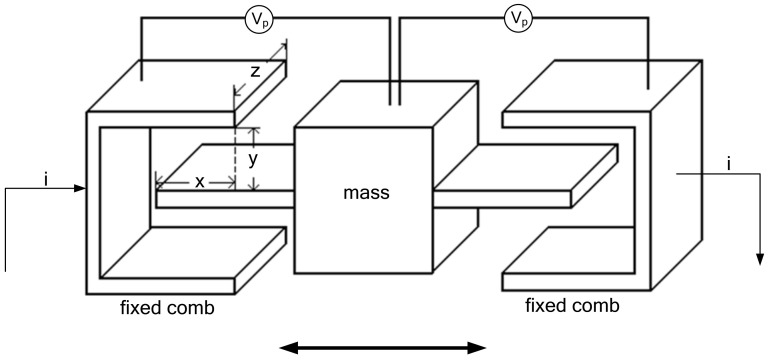
Structure of drive-sensing combs.

Figure 2 depicts the sensitive combs’ driving schematic. As the proof mass moves, the capacitance between the fixed combs and the proof mass changes accordingly. If a DC voltage is applied between the electrodes of the fixed combs and the proof mass, the current can be calculated as

(1) $$i=\frac{dQ}{dt}=\frac{d(CV)}{dt}=C\frac{dV}{dt}+V\frac{dC}{dt}$$

Figure 2 shows the DC voltage between the fixed comb teeth and the electrodes on the mass block. If the first term to the right of Equation (1) is zero, the number of mass blocks is

(2) $$i=N_{2}V_{p}\frac{dC}{dt}=N_{2}V_{p}\frac{dC}{dx}\frac{dx}{dt}=N_{2}V_{p}\varepsilon \frac{z}{y}\frac{dx}{dt}$$

Equation (2) demonstrates that the output current of the micromechanical gyroscope’s driving sensitive comb tooth in the *x*-axis direction is directly proportionate to the speed.

### 2.2. Sense Mode

The vibration of the micromechanical gyroscope on the *x*-axis results in the generation of Coriolis force acceleration on the *y*-axis via the angular velocity in the *z*-axis. When the sense combs vibrate on the *y*-axis, the equivalent capacitance changes [21]. The principle underlying the detection of angular velocity is based on the detection of alterations in the equivalent capacitance on the *y*-axis. The amplitude of capacitance vibration is directly proportional to the input angular velocity.

**Figure 3:**
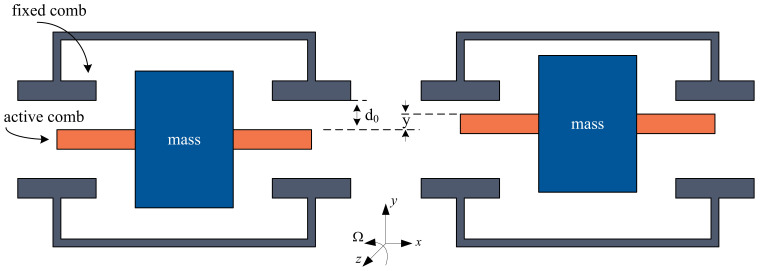
Structure of sense combs.

The proof mass experiences no Coriolis force and the sense comb remains central when the external input angular velocity is zero. In addition, the distances between the fixed combs on the upper and lower sides are equal, and thus the values of C1 and C2 are also equal. At this point, the sensor is in the zero position, as depicted in the left portion of Figure 3. However, when the external angular velocity signal is not zero, the proof mass experiences Coriolis force, causing the mass block to shift along the detection axis. This movement leads to variations in the capacitance values on both sides, as illustrated on the right side of Figure 3.

The dynamic equation of sense direction is

(3) $$M_{s}\frac{d^{2}y}{dt^{2}}+\lambda_{s}\frac{dy}{dt}+K_{s}y=2B_{d}M_{s}\Omega_{0}\omega \cos\omega_{i}t\cos(\omega t+\varphi_{d})$$

where the proof mass is represented by $M_{s}$ and $\lambda_{s}$ is the damping force coefficient acting in the detection axis direction. $K_{s}$ is the elastic coefficient in the same direction; *y* is the vibration displacement detected in the detection axis direction.

To obtain the angular velocity signal with the maximum amplitude, we selected $\cos(\omega t+\frac{\varphi_{s1}+\varphi_{s2}}{2}+\varphi_{d})$ as the reference signal for demodulation, and the output after demodulation was

(4) $$\begin{array}{l} y_{1}(t)=\frac{1}{2}(B_{s1}+B_{s2})\cos(\omega_{i}t+\frac{\varphi_{s2}-\varphi_{s1}}{2})\{1+\cos[2(\omega t+\frac{\varphi_{s1}+\varphi_{s2}}{2}+\varphi_{d})]\}\\ +\frac{1}{2}(B_{s1}-B_{s2})\sin(\omega_{i}t+\frac{\varphi_{s2}-\varphi_{s1}}{2})\sin[2(\omega t+\frac{\varphi_{s1}+\varphi_{s2}}{2}+\varphi_{d})] \end{array}$$

The frequency of the driving signal for the micromechanical gyroscope is typically several hundred times greater than the input angular velocity frequency. This discrepancy allows for the simplification of the signal, represented in Formula (4). A low-pass filter can effectively remove the higher-frequency component present in the signal expression.

(5) $$y_{2}(t)=\frac{1}{2}(B_{s1}+B_{s2})\cos(\omega_{i}t+\frac{\varphi_{s2}-\varphi_{s1}}{2})$$

The frequency of the output signal is equal to that of the angular velocity signal. Furthermore, there is direct proportionality between the amplitude of the output signal and the angular velocity signal. Such a correlation enables the measurement of the angular velocity signal by detecting the corresponding amplitude and frequency of the output signal.

### 2.3. Closed-Loop Driving Stability Analysis

**Figure 4:**
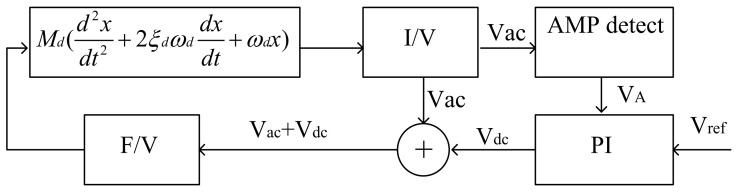
Closed-loop self-excited drive system.

The block diagram of the closed-loop self-excited drive system is depicted in Figure 4. A positive feedback loop is formed by the I/V converter, adder, F/V converter and gyroscope structure, resulting in the self-excited oscillation of the gyro. Meanwhile, a feedback control loop is established by the peak detection circuit and the proportional integral (PI) controller to ensure a stable driving amplitude. The PI controller regulates the amplitude of vibration while the F/V converter stabilizes the amplitude of the Vac. The gyroscope in the *X*-axis direction is

(6) $$M_{d}(\frac{d^{2}x}{dt^{2}}+2\xi_{d}\omega_{d}\frac{dx}{dt}+\omega_{d}^{2}x)=KV_{dc}\frac{dx}{dt}$$

where *M_d_* is the equivalent mass in the drive axis, *ω_d_* is the natural frequency of the drive mode, *ξ_d_* is the damping ratio and *K* is the loop gain.

The peak detection circuit comprises an amplitude detection circuit and a low-pass filter. The electrical differential equation of the peak detection circuit is represented by Equation (3), where *K*_1_ denotes the coefficient of the amplitude detection circuit, *V_A_* represents the output signal of the peak detection circuit and $\tau_{1}$ is the time constant of the low-pass filter.

(7) $$|K_{1}V_{ac}|=V_{A}+\tau_{1}\frac{dV_{A}}{dx}$$

The PI controller is composed of a proportional amplification component and an integral component. The electrical differential equation for the PI controller is represented by Equation (8). Notably, *K*_2_ serves as both the coefficient for the proportional part and the integral time constant.

(8) $$-\frac{dV_{dc}}{dx}=K_{2}\frac{dV_{A}}{dx}+\tau_{2}(V_{A}-V_{ref})$$

Equations (6)–(8) form a system of differential equations, which describes a nonlinear system. Solving the differential equations allows for the determination of the initial vibration condition of the system. The solution system’s characteristic root is

(9) $$S=-\frac{1}{2\tau_{1}}(1\pm \sqrt{1-2\tau_{1}(\frac{KV_{ref}}{M_{d}}-\xi_{d}\omega_{d})})$$

So, the initial vibration condition of the system is

(10) $$\frac{KV_{ref}}{M_{d}}-\xi_{d}\omega_{d}>0$$

(11) $$\tau_{1}\tau_{2}<K_{2}$$

The driving force of the gyroscope, as defined by Formula (11), must exceed the damping force. The stability of a nonlinear system can be determined by analyzing the real part of its characteristic roots; a negative real part indicates stability with a consistent post-vibration amplitude. When the conditions required to initiate vibration are met, a stable vibration amplitude can be achieved.

When $2\tau_{1}(\frac{KV_{ref}}{M_{d}}-\xi_{d}\omega_{d})$ > 1, overshoot and oscillation will be generated, and increasing $\tau_{1}$ will increase the duration of the overshoot and oscillation phenomenon. The oscillation degree will be deepened, resulting in the instability of the system. When $2\tau_{1}(\frac{KV_{ref}}{M_{d}}-\xi_{d}\omega_{d})$ < 1, the amplitude of the gyro-driven signal will converge to the stable value at the speed of $2\tau_{1}$. Increasing *KV_ref_* can shorten the starting time.

## 3. Analysis of Automatic Gain Control Circuit

**Figure 5:**
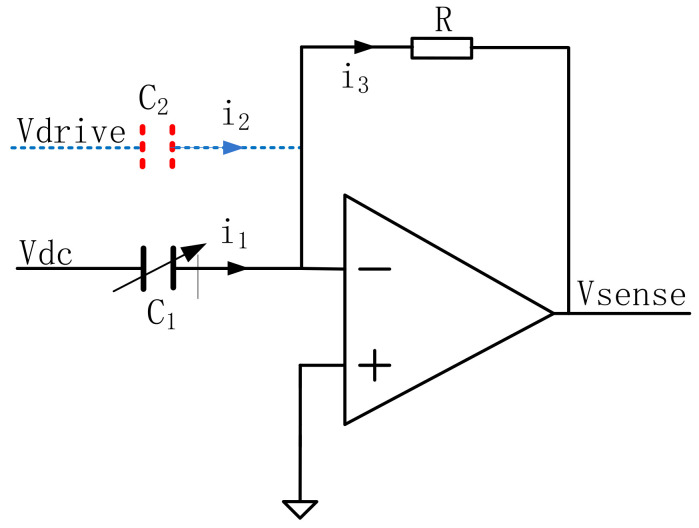
Schematic diagram of driving signal coupling.

The principle is shown in Figure 5. Capacitor C_2_ is the parasitic capacitance between the driving end of the gyro structure and the driving detection end. The driving voltage *V*_drive_ is converted into current *i*_2_ through the capacitor C_2_. The frequency of this current is the same as the frequency of the driving voltage. The phase difference in the current is 90°, and the expressions of *i*_1_ and *i*_2_ can be expressed as follows:

(12) $$i_{1}=A\sin\omega t$$

(13) $$i_{2}=B\cos\omega t$$

The feedback current *i*_3_ of the charge amplifier is as follows:

(14) $$i_{3}=i_{1}+i_{2}=A\sin\omega t+B\cos\omega t=\frac{1}{\sqrt{A^{2}+B^{2}}}\sin(\omega t+\theta )$$

(15) $$\theta =\mathrm{arctg}\frac{B}{A}$$

The parasitic capacitance *C*_2_ causes the phase and amplitude of the feedback current of the charge amplifier at the driving end to change. The phase difference between the feedback current and *i*_1_ changes the angle, and the angle value is determined by the ratio of the parasitic capacitance to the differential capacitance. According to the classic oscillation principle, the closed-loop self-excited drive requires that the phase difference between the drive signal and the charge amplifier meet certain conditions and must be smaller than a predetermined low value [22,23]. Exceeding this value will cause the oscillation phase condition to be inconsistent and the circuit will not oscillate. Therefore, to reduce the impact of parasitic capacitance *C*_2_, the automatic gain control circuit needs to decrease the loop gain or choose a high *Q* value gyroscope structure [24,25].

From the perspective of sensor head design, good packaging, preferably vacuum packaging, is required. Therefore, the head of the normal temperature and pressure package should be designed using a frequency modulation scheme. After adopting this driving scheme, the frequency of the driving voltage changes, differing from the frequency of the sensitive current after square wave modulation. In this way, the frequency separation between the AC driving voltage signal and the sensitive current signal can be realized, the problem that the closed-loop system cannot self-oscillate can be solved and the environmental interference caused by the large loop gain can also be reduced.

## 4. Frequency Modulation Drive Circuit

**Figure 6:**
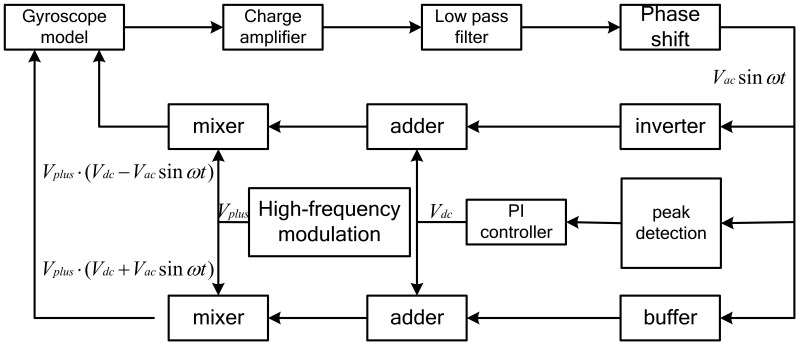
Frequency modulation drive circuit.

The principle of the frequency modulation drive circuit is shown in Figure 6. The sensitive current is converted into a voltage signal through the charge amplifier. The phase difference between the voltage signal and the sensitive current signal is 90°. The phase shift of the pre-stage charge amplifier is offset by the post-stage 90° phase shifter to meet the phase condition of the closed-loop drive. The signal is amplified by a first-stage amplifier circuit to output three signals. All the way through the peak detection circuit, PI controls the DC voltage output. After the other two paths pass through the follower and the inverter, the AC signals are obtained. The three-way signals pass through two adders to obtain signals, are multiplied by the high-frequency carrier and then they output the drive signals of the micromechanical gyroscope to achieve closed-loop driving.

### 4.1. Charge Amplifier

**Figure 7:**
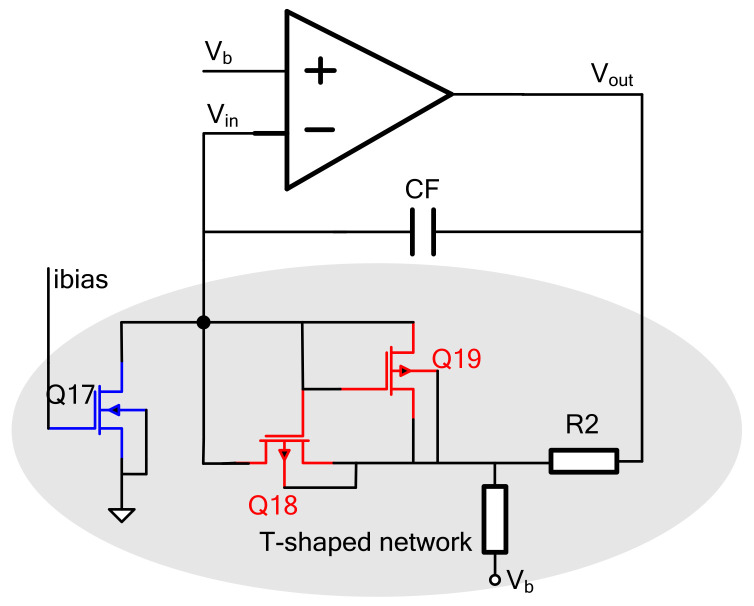
Charge amplifier.

Figure 7 is a circuit structure diagram of a charge amplifier based on a folded cascode three-stage operational amplifier. The equivalent input noise density of this amplifier is as follows:

(16) $$V_{n}^{2}=\frac{16kT}{3}(\frac{1}{g_{m2}}+\frac{g_{m5}+g_{m8}}{g_{m2}^{2}})$$

The charge amplifier uses a T-shaped network structure to increase the transimpedance gain, as shown in Figure 4. Transistors Q17~Q19 and resistors R1 and R2 form a T-shaped resistor network larger than 107 Ω. Using Q17 and Q19 allows Q18 to achieve a smaller VGS and reduces the W/L of Q18; so, Q18 is in the linear region. Its equivalent resistance exceeds 1 MΩ and is proportional to the bias resistance R in the bias circuit, without significant changes in time and temperature. Capacitor CF is about 0.3 pF, which can improve the stability of the system. The equivalent resistance is shown in Formula (6):

(17) $$R_{eq}=R_{M}(1+\frac{R_{2}}{R_{1}})+R_{2}$$

*R_M_* is the equivalent resistance of Q18. If *R_M_* is greater than 1 MΩ, it will become the main noise source of the charge amplifier. The charge amplifier output signal-to-noise ratio *SNR* determined by the equivalent resistance of the transistor can be expressed as follows:

(18) $$SNR=\sqrt{\frac{I_{IN}^{2}R_{eq}}{4KT(R_{2}/R_{1}+1)}}\approx \sqrt{\frac{I_{IN}^{2}R_{M}}{4KT}}$$

where *I_IN_* is the charge amplifier input current:

(19) $$I_{IN}=\frac{\partial C}{\partial t}V_{b}=C_{0}V_{b}\omega \sin\omega t$$

*C*_0_ is the maximum variation in the sensitive capacitance at the drive end, *ω* is the resonant frequency and *V_b_* is the forward bias voltage.

### 4.2. Phase Shifter Circuit

**Figure 8:**
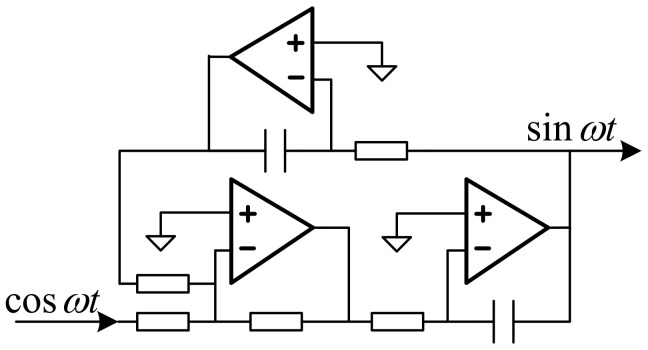
Phase shifter circuit.

The phase shifter circuit consists of an adder and two integrators, as shown in Figure 8. It can carry out the 90° phase shift of the input signal and realize the conversion of DC bias voltage. The resistance values of the three resistors of the adder are equal, and the integrator is divided into a feedback integrator and a feedforward integrator. After the latter achieves a 90° phase shift, the output current value is fed back to the adder via the feedback integrator.

### 4.3. PI Controller Circuit

**Figure 9:**
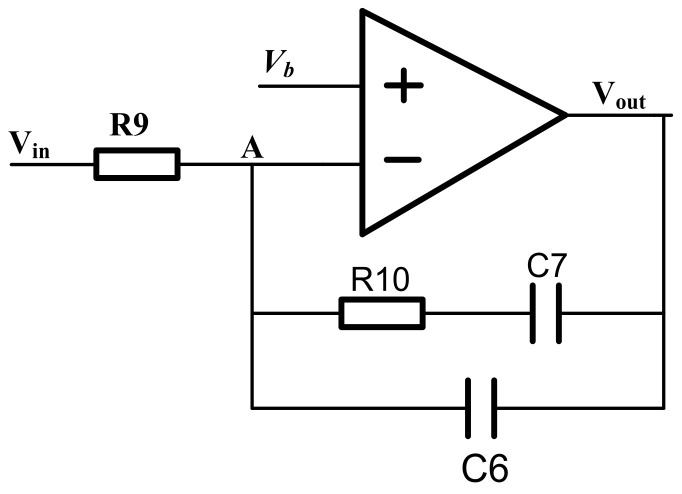
PI controller.

The PI controller circuit adopts the structure shown in Figure 9, in which the operational amplifier and capacitor C6 form an integrator, the output of the peak detection circuit after passing through the filter is connected to the negative input terminal of the operational amplifier through the resistor R9 and its positive input terminal is connected to the DC comparison voltage *V_r_*_ef_. The PI controller adjusts the DC part of the driving signal. The working mode can be analyzed using a DC comparator. Due to the “virtual short” of the operational amplifier, the voltage of node A is equal to *V_r_*_ef_. If the voltage difference between node *V*_out_ and node A is the same as when it is zero, there is a constant current flowing through the resistor R9, and the output node voltage changes after the current is integrated by C6. When the integrated current value flowing through C6 is zero, the system enters a steady state and the output node voltage is equal to the voltage of node A.

### 4.4. Mixer Circuit

To avoid the influence of the offset voltage of the operational amplifier in the mixer, the temperature coefficient of the offset voltage should be reduced. The topology of the three-stage operational amplifier is adopted, the second-stage capacitor is compensated and the switch unit adopts a 6 MOS structure, which effectively eliminates clock feedthrough and charge injection, as well as lower on-resistance.

**Figure 10:**
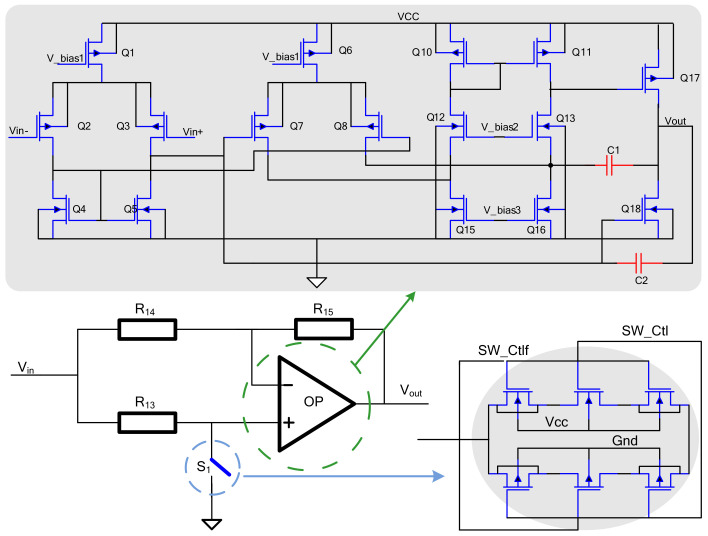
Mixer circuit.

As shown in Figure 10, one end of the switch S1 is connected to the positive end of the operational amplifier, while the other end is connected to the reference voltage *V*_ref_, and is turned on and off by a square wave signal. The signal at the positive input of the mixer can be expressed as follows:

(20) $$U_{2}(t)=\left\{\begin{array}{cc} V_{ref} & nT_{C}<t<\left(n+\frac{1}{2}\right)T_{C}\\ Us(t) & \left(n+\frac{1}{2}\right)T_{C}<t<\left(n+1\right)T_{C} \end{array}\right.$$

If the resistors R14 and R15 are equal, then the output waveform of the mixer is as follows:

(21) $$U_{o}(t)=-U_{s}(t)+2U_{2}(t)$$

Substituting Formula (9) into (10), Expression (11) can be obtained:

(22) $$U_{o}(t)=\left\{\begin{array}{cc} 2V_{ref}-U_{s}(t) & nT_{C}<t<\left(n+\frac{1}{2}\right)T_{C}\\ U_{s}(t) & \left(n+\frac{1}{2}\right)T_{C}<t<\left(n+1\right)T_{C} \end{array}\right.$$

## 5. Results

Table 1 shows the structural parameters of the silicon gyroscope employed in this paper.

**Figure 11:**
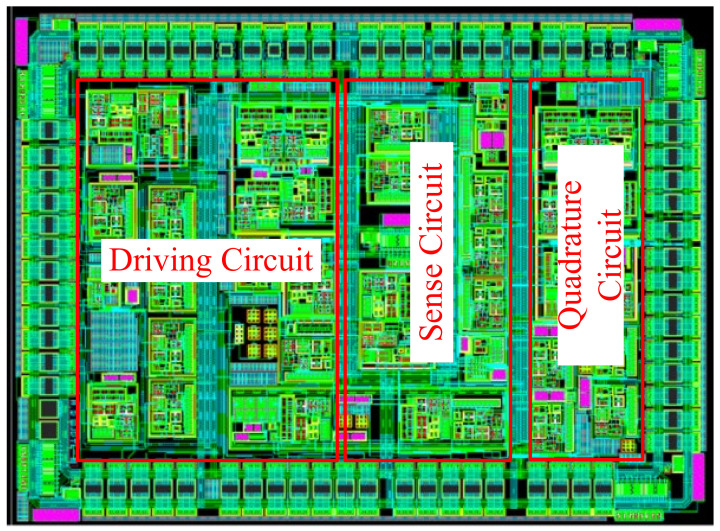
Layout of closed-loop micromachined gyroscope interface ASIC.

The monolithic integrated design of the MEMS interface circuit is based on 0.5 μm 18 V CMOS process technology. To reduce the low-frequency 1/f noise and thermal noise of the charge amplifier, the charge amplifier input transistor uses a large aspect ratio. To prevent the AC signal of the driving circuit affecting the sense circuit, the driving and the sense circuit module become separate modules. Meanwhile, a grounded metal wire is added between the driving circuit and the sense circuit, reducing the interference of the driving signal in the sensitive circuit. In addition, the charge amplifier input tube used in the interface ASIC provides a fully symmetrical layout, which can reduce the amplifier’s offset voltage. The interface circuit chip area is about 5.05 mm × 3.7 mm, and the overall interface circuit includes the gyroscope drive circuit, the sense circuit and the quadrature circuit. The interface ASIC contains 818 transistors, 54 resistors, 60 capacitors and 5 diodes. The layout of the MEMS gyroscope interface ASIC is shown in Figure 11. The gyroscope interface ASIC operates at ±9 V supply voltage and its power consumption is 360 mW.

### 5.1. System-Level Modeling and Simulation

**Figure 12:**
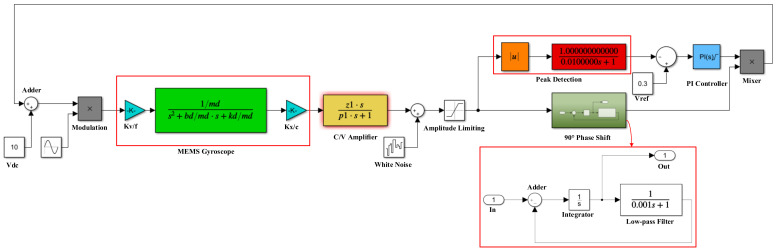
Driving circuit system-level simulation model diagram.

The performance of the driving circuit is also one of the key factors limiting the performance of the gyroscope system. The MEMS gyroscope interface circuit adopts the automatic gain control module to achieve stable amplitude control of the driving voltage, ensuring the self-excited oscillation of the gyroscope under normal temperature and a low Q value. The driving circuit allows for complete closed-loop self-excited oscillation, and the sense circuit can detect the input angular velocity information. After the driving circuit stabilizes the self-excited amplitude, the sense circuit can complete the demodulation and output of the angular velocity signal. Therefore, it is crucial for the MEMS gyroscope’s driving circuit to achieve self-excited oscillation. In this work, a system-level simulation model of the gyroscope driving loop was established using SIMULINK, as shown in Figure 12.

**Figure 13:**
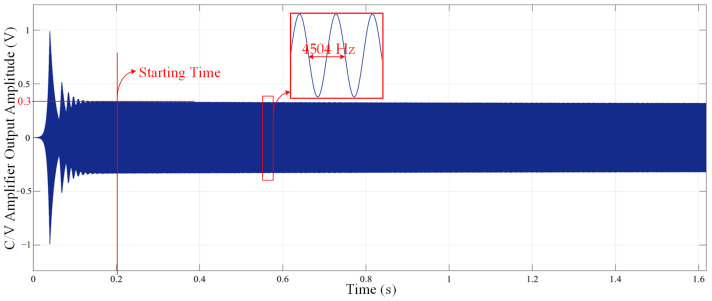
The simulation diagram of the self-excited start of the driving circuit of the MEMS gyroscope.

The automatic gain module ensures that the driving circuit has stable amplitude and is quick to start, allowing the gyroscope system to quickly enter into operation. By changing the value of the set reference voltage, the oscillation amplitude of the gyroscope drive circuit can be altered. Figure 13 shows the simulation diagram of the self-excited start of the driving circuit of the MEMS gyroscope. The simulation results show that the driving circuit completes the self-excitation oscillation in about 0.2 s, and the resonant frequency of the driving voltage signal is 4504 Hz. Compared with the non-self-excited oscillation method, the system does not need to readjust the locking frequency and phase when the resonant frequency of the gyroscope changes. Therefore, the closed-loop self-excited drive scheme based on the secondary demodulation principle is more adaptable and consumes less hardware resources.

### 5.2. Transistor-Level Simulation Analysis

**Figure 14:**
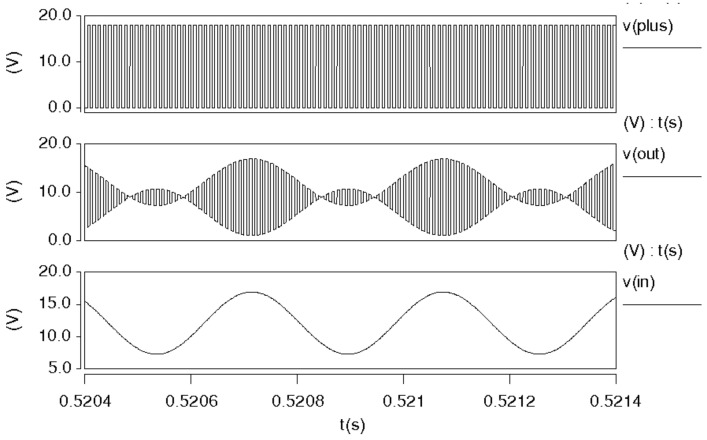
Simulated result of mixer.

Figure 14 is the overall simulation result of the mixer. As shown in the figure, the output signal of the mixer is an envelope signal, including the signal and the switch signal. The outside of the envelope is a sinusoidal signal with the same frequency, and the signal inside the envelope is at the same frequency as the square wave signal. The amplitude of the square wave signal in the figure is double *V*_ref_.

**Figure 15:**
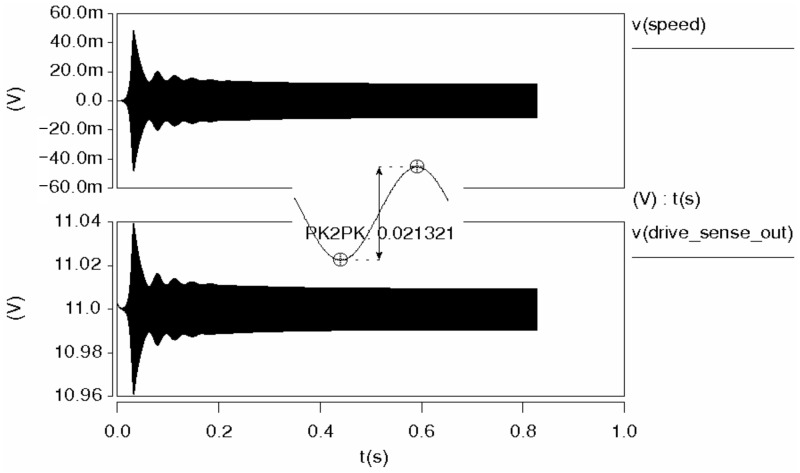
Transient response of closed-loop driving system.

The overall transient simulation results of the closed-loop drive system are shown in Figure 15. V(drive_sense_out) is the detection signal of the micromachined gyroscope in the driving direction, and its vibration speed is represented by V(speed). The signal of the driving loop based on frequency modulation reaches a stable state soon after the oscillation. The stabilization time is approximately 200 ms, the detection signal amplitude is about 21.32 mV and the amplitude does not change over time after stabilization. Therefore, the designed frequency-based demodulation circuit successfully stabilizes the vibration.

**Figure 16:**
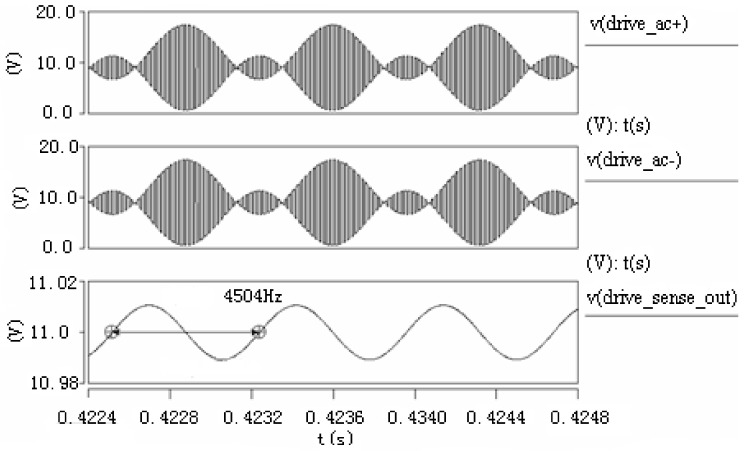
Transient response of micromachined gyroscope driving circuit.

The waveform diagram of the driving signal of the micromachined gyroscope in the steady state is shown in Figure 16. V(drive_ac+) and V(drive_ac-) with opposite phases are the frequency-modulated driving voltage signals loaded on the driving electrodes of the micromechanical gyroscope, respectively. V(drive_sense_out) is a drive detection signal. The detection signal frequency in the driving direction of the micromachined gyroscope is equal to its natural frequency, which is determined via its frequency selection characteristics. The stable average frequency is 4504 Hz and the frequency deviation is 1 Hz. Based on the mean square deviation of the simulation data after the system is stable, the frequency jitter is 0.0221 Hz.

### 5.3. Experimental Test

**Figure 17:**
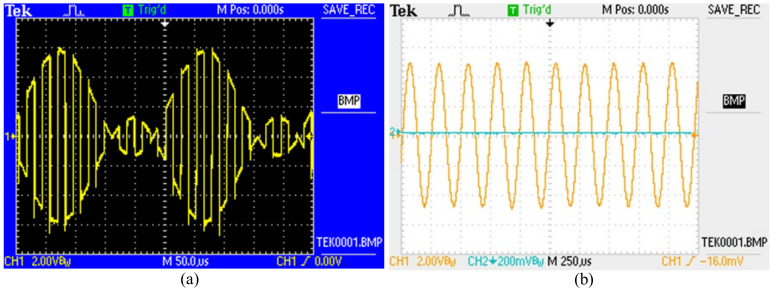
Experimental results of driving circuit of the MEMS gyroscope: (**a**) test results of electrostatic driving modulation signal; (**b**) test results of driving signal time domain.

In order to verify the feasibility of the drive circuit design scheme of the MEMS gyroscope, the closed-loop self-excited oscillation of the gyroscope interface ASIC chip was tested experimentally. Figure 17a shows the test result of the electrostatic force drive modulation signal of the MEMS gyroscope. The drive force signal was frequency-modulated, which is consistent with the designed driving force signal. Figure 17b presents the time domain test chart of the MEMS gyroscope drive signal, and the test results show that the designed driving circuit can achieve the closed-loop driving function.

**Figure 18:**
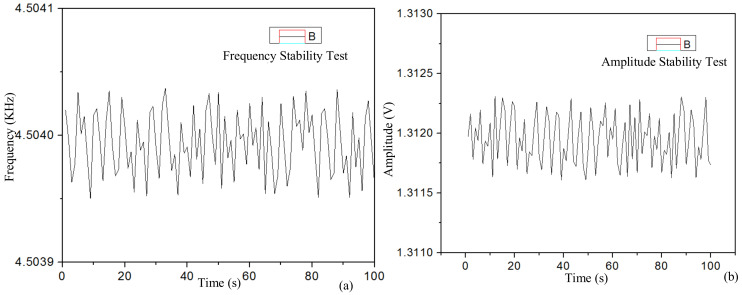
Test results of driving circuit stability of the MEMS gyroscope: (**a**) driving displacement amplitude stability; (**b**) drive signal frequency stability.

Figure 18a shows the test results of the driving vibration signals of ASIC, the driving circuit interface of the MEMS gyroscope. One point was picked every 1 s, and one hundred points were picked, respectively, for each test experiment. The experimental results demonstrate that the interface ASIC can realize closed-loop self-excited driving with stable amplitude and frequency under normal pressure. Frequency stability was measured using the frequency channel of a multimeter (Agilent34410A). The resonant frequency was 4.504 kHz, the frequency fluctuation was less than 0.1 Hz and the relative stability was about 0.0025%. The stability of the driving displacement amplitude was tested using the voltage channel of a multimeter. The relative stability of the driving displacement amplitude was about 0.0050%.

Table 2 compares the performance of various driving circuits for the MEMS gyroscope. The self-excited oscillation driving circuit of this design, which was based on the secondary demodulation principle, was monolithically integrated using 0.5 μm CMOS process technology, which has a high degree of integration. By comparing the measured results of the method of optimizing phase noise with the experimental results of the scheme without considering phase noise optimization, Table 2 shows that the self-excited oscillation driving circuit designed using optimized phase noise improved the driving stability. The MEMS gyroscope interface circuit designed in this work achieved monolithic integration and good overall performance.

## 6. Conclusions

In this paper, a self-excited oscillation driving interface circuit based on the principle of quadratic demodulation was proposed. This circuit is suitable for low-Q gyroscopes under normal temperature and pressure. A self-excited closed-loop driving circuit consisting of a phase shifter, PI controller and mixer was designed, and it can precisely and stably control the mechanical sensitive element. This interface circuit can eliminate the influence of the drive electrode on the displacement electrode and reduce the frequency jitter, improving the overall performance of the gyroscope. Thus, the gyro’s driving interface ASIC is monolithically integrated using standard 0.5 μm CMOS process technology. Then, the system-level modeling and simulation of the gyroscope driving loop were carried out using SIMULINK, and the frequency and amplitude stability of the gyroscope driving circuit were tested and verified. The results show that the relative stability of the drive circuit frequency stability was about 0.0025%, and the frequency fluctuation was less than 0.1 Hz. The relative stability of the driving displacement amplitude was about 0.0050%. The MEMS gyroscope driving circuit test experiment verifies the correctness of the interface ASIC chip design scheme, and the gyroscope driving circuit can achieve a good, stable amplitude of self-excited oscillation.

## Figures and Tables

**Table 1:** Structural parameters of the silicon gyroscope.

Parameter		Unit
Drive mass *M_d_*	2.1493	Kg × 10^−7^
Damping coefficient	2.1247	*λ_d_* × 10^−5^
Elastic coefficient	1.7219	*Kd* × 10^2^
Central capacitance	0.9445	pF
Resonant frequency	4.5047	KHz
Quality factor	205.7238	

**Table 2:** Comparison of the MEMS gyroscope’s driving circuit stability.

Ref	Frequency (kHz)	Frequency Stability	Amplitude Stability
[26]	-	0.0290%	0.0470%
[27]	54.76	0.003%	0.0025%
This work	4.504	0.0025%	0.005%

## Data Availability

The data that support the findings of this study are available from the corresponding author upon reasonable request.

## References

[1] Li X., Wang W., Wang S., Peng Y., Jin X. (2019). Status and development trend of MEMS inertial sensors. Meas. Technol..

[2] Yi J., Jiang N., Zhuang S., Guo S., Zhang J. (2018). Status and development of MEMS solid-state fluctuating gyro resonators. Micro Nanoelectron..

[3] Yang B., Wang S.R., Li K.Y., Zhu X., Cao H. (2010). Silicon tuning gyroscope tuned by negative stiffness effect. Opt. Precis. Eng..

[4] Hou Z., Kuang Y., Ou F., Xu Q., Miao T., Xiao D., Wu X. (2021). A quadrature compensation method to improve the performance of the butterfly vibratory gyroscope. Sens. Actuators A Phys..

[5] Jeong C., Seok S., Lee B., Kim H., Chun K. (2004). A study on resonant frequency and Q factor tunings for MEMS vibratory gyroscopes. J. Micromech. Microeng..

[6] Miao T., Hu X., Zhou X., Wu X., Hou Z., Xiao D. A Million-order Effective Quality Factor MEMS Resonator by Mechanical Pumping. Proceedings of the 2020 IEEE International Symposium on Inertial Sensors and Systems (INERTIAL).

[7] Xu P., Si C., He Y., Wei Z., Jia L., Han G., Ning J., Yang F. (2021). A Novel High-Q Dual-Mass MEMS Tuning Fork Gyroscope Based on 3D Wafer-Level Packaging. Sensors.

[8] Xu P., Wei Z., Guo Z., Jia L., Han G., Si C., Ning J., Yang F. (2021). A Real-Time Circuit Phase Delay Correction System for MEMS Vibratory Gyroscopes. Micromachines.

[9] Keymeulen D., Peay C., Foor D., Trung T., Bakhshi A., Withington P., Yee K., Terrile R. Control of MEMS Disc Resonance Gyroscope (DRG) using an FPGA Platform. Proceedings of the Aerospace Conference.

[10] Goto K., Harada S., Hata Y., Ito K., Wado H., Cho J.Y., Najafi K. High Q-Factor Mode-Matched Silicon Gyroscope with a Ladder Structure. Proceedings of the 2020 IEEE International Symposium on Inertial Sensors and Systems (INERTIAL).

[11] Elwell J.M. Micromechanical Inertial Swors for Commercial and Military Applications. Proceedings of the 50th Annual Meeting of The Institute of Navigation.

[12] Joachim D., Lin L. Selective polysilicon deposition for frequency tuning of MEMS resonators. Proceedings of the Micro Electro Mechanical Systems, 2002, the Fifteenth IEEE International Conference on IEEE.

[13] Wang Z., Fei J. (2022). Fractional-Order Terminal Sliding-Mode Control Using Self-Evolving Recurrent Chebyshev Fuzzy Neural Network for MEMS Gyroscope. IEEE Trans. Fuzzy Syst..

[14] Lv R.S., Fu Q., Yin L., Gao Y., Bai W., Zhang W.B., Zhang Y.F., Chen W.P., Liu X.W. (2019). An Interface ASIC for MEMS Vibratory Gyroscopes with Nonlinear Driving Control. Micromachines.

[15] Li X.Y., Hu J.P., Liu X.W. (2018). A high-performance digital interface circuit for a high-Q micro-electromechanical system accelerometer. Micromachines.

[16] Huang F., Liang Y. (2017). Analysis and design of the system of a total digital Si-gyroscope. Int. J. Mod. Phys. B.

[17] Ahn C.H., Ng E.J., Hong V.A., Yang Y.S., Lee B.J., Flader I., Kenny T.W. (2015). Mode-matching of wineglass mode disk resonator gyroscope in (100) single crystal silicon. J. Microelectromech. Syst..

[18] Sonmezoglu S., Alper S.E., Akin T. (2014). An automatically mode-matched MEMS gyroscope with wide and tunable bandwidth. J. Microelectromech. Syst..

[19] Balachandran G.K., Petkov V.P., Mayer T., Balslink T. (2016). A 3-axis gyroscope for electronic stability control with continuous self-test. IEEE J. Solid-State Circuits.

[20] Lv R.S., Chen W.P., Yin L., Fu Q., Liu X.W., Yan J.M. (2018). A closed-loop SD modulator for micromechanical capacitive sensors. IEICE Electron. Express.

[21] Lv R.S., Chen W.P., Liu X.W. (2018). A high-dynamic-range switched-capacitor sigma-delta ADC for digital micromechanical vibration gyroscopes. Micromachines.

[22] Zhao Y., Zhao J., Wang X., Xia G.M., Shi Q., Qiu A.P., Xu Y.P. (2018). A sub-0.1 degrees/h bias-instability split-mode MEMS gyroscope with CMOS readout circuit. IEEE J. Solid-State Circuits.

[23] Feng C., Liu D., Ma H., Qing C., Li H., Feng L. (2021). Design, fabrication and test of transmissive Si_3_N_4_ waveguide ring resonator. IEEE Sens. J..

[24] Yang C., Li H.S., Xu L., Zhu K. (2016). Low-frequency modulated excitation-based automatic mode-matching technique for silicon micro gyroscope. Chin. J. Inert. Technol..

[25] Bu F., Peng Y., Xu D., Zhao H. (2018). A MEMS gyro mode-matching method combining quadrature control and phase detection. Chin. J. Inert. Technol..

[26] Zhixiong Z., Lihui F., Yu-nan S. (2011). Temperature Modeling and Compensation of Double h Quartz Tuning Fork Gyroscope. Procedia Eng..

[27] Zhang H., Chen W.P., Liang Y. (2023). An Interface ASIC Design of MEMS Gyroscope with Analog Closed Loop Driving. Sensors.

